# Determining risk and predictors of head and neck cancer treatment-related lymphedema: A clinicopathologic and dosimetric data mining approach using interpretable machine learning and ensemble feature selection

**DOI:** 10.1016/j.ctro.2024.100747

**Published:** 2024-02-28

**Authors:** P. Troy Teo, Kevin Rogacki, Mahesh Gopalakrishnan, Indra J Das, Mohamed E Abazeed, Bharat B Mittal, Michelle Gentile

**Affiliations:** a Department of Radiation Oncology, Northwestern Memorial Hospital, Robert H. Lurie Comprehensive Cancer Center, Northwestern University Feinberg School of Medicine, 251 E. Huron St, Galter Pavilion LC-178, IL 60611. Chicago, United States; b Department of Radiation Oncology, University of Pennsylvania, Pennsylvania Hospital, 800 Spruce Street, Philadelphia, PA 19107, United States

**Keywords:** Head and neck cancer, Oropharyngeal cancer, Lymphedema, Early onset lymphedema, Machine learning, Radiation dose response relationship, Assessments, risk, Interpretable AI, Explainable AI

## Abstract

**Background and purpose:**

The ability to determine the risk and predictors of lymphedema is vital in improving the quality of life for head and neck (HN) cancer patients. However, selecting robust features is challenging due to the multicollinearity and high dimensionality of radiotherapy (RT) data. This study aims to overcome these challenges using an ensemble feature selection technique with machine learning (ML).

**Materials and methods:**

Thirty organs-at-risk, including bilateral cervical lymph node levels, were contoured, and dose-volume data were extracted from 76 HN treatment plans. Clinicopathologic data was collected. Ensemble feature selection was used to reduce the number of features. Using the reduced features as input to ML and competing risk models, internal and external lymphedema prediction capability was evaluated with the ML models, and time to lymphedema event and risk stratification were estimated using the risk models.

**Results:**

Two ML models, XGBoost and random forest, exhibited robust prediction performance. They achieved average F1-scores and AUCs of 84 ± 3.3 % and 79 ± 11.9 % (external lymphedema), and 64 ± 12 % and 78 ± 7.9 % (internal lymphedema). Predictive ML and risk models identified common predictors, including bulky node involvement, high dose to various lymph node levels, and lymph nodes removed during surgery. At 180 days, removing 0–25, 26–50, and > 50 lymph nodes increased external lymphedema risk to 72.1 %, 95.6 %, and 57.7 % respectively (p = 0.01).

**Conclusion:**

Our approach, involving the reduction of HN RT data dimensionality, resulted in effective ML models for HN lymphedema prediction. Predictive dosimetric features emerged from both predictive and competing risk models. Consistency with clinicopathologic features from other studies supports our methodology.

## Highlights

• External & internal lymphedema prediction for entire HN & oropharyngeal cohorts.
• Pioneering inclusion of dosimetric predictors offers holistic lymphedema prognosis.
• Successful reduction of high-dimensional RT dataset via ensemble feature selection.
• Aggregating feature importance from models reduces bias, improves interpretability.
• Shared lymphedema prognostic features with other studies give model credibility.

## Introduction

Lymphedema is one of the most common treatment-related side effects after radiotherapy (RT) of head and neck (HN) cancer that negatively impacts the quality of life [1], [2], [3]. It has been estimated that more than two-thirds of HN cancer patients will develop some form of lymphedema [1]. Numerous studies have focused on generating measurements and understanding the etiology and progression of HN lymphedema [4], [5], [6], [7], [8], [9]. While one recent study examined the clinicopathologic data for predictors of HN lymphedema [10]; another study looked at dosimetric factors associated with HN lymphedema following treatment for nasopharyngeal carcinoma [11], no prior study has comprehensively evaluated both patient, tumor, treatment, and dosimetric variables for a range of HN cancer types. Although dosimetric data have been evaluated for possible causes of other radiation-induced toxicities, such as lymphedema from breast RT [12], late dysphagia after HN RT [13], radiation-induced xerostomia [14], and brain stem injury [15] after HN RT, a common challenge identified in these HN studies involves the high dimensionality and multicollinearity of the dosimetric data [11], [12], [13], [14], [15], [16]. Both issues could lead to the problem of over-fitting the model, making it potentially difficult to identify features that are most predictive of lymphedema consistently [15].

Various techniques have been implemented to model the association of radiation-induced toxicity with dosimetric and clinical features. The classical statistical method tends to rely on linear-regression-like models to iteratively prune/add variables from an existing dataset until an optimal score (e.g., Akaike Information Criterion, AIC) is obtained for the model [10], [11], [12], [15]. It uses statistical significance testing (e.g., p-value) to infer the feature's prognostic value. Testing on unseen data is often not involved. The machine learning (ML) approach focuses on developing a generalizable model to capture the data pattern and test its predictability using unseen out-of-sample data [16], [17]. Despite the inference versus prediction nature of the model [18], [19], outcomes from both approaches are prone to a certain degree of model-specific biasness [18]. To overcome the problems of model biasness, high dimensionality, and multicollinearity in data, a robust machine learning approach with an appropriate feature selection strategy must be employed.

Recent studies using ensemble feature selection have shown improved stability of features selected from high dimensional, low sample size (HDLSS) datasets [20], [21], [22], [23]. Although the ensemble feature selection approach has been used in bioinformatics [21], [22] and large medical datasets [23], it has rarely been applied to RT dosimetric datasets. This multi-stage selection approach uses a filter, wrapper, and embedded methods–combinations of inference and predictive techniques coupled with a ranking mechanism to determine features of relevance for the predicted outcome. One ranking mechanism that is of increasing interest is feature importance [24]. Instead of creating new abstractions of features that are difficult to interpret (e.g., principal components), feature importance enhances the interpretability of the feature selection process by retaining the original representation of the features. While feature importance is model-specific, aggregating feature importance from different models to provide a consensus of the most commonly selected feature helps mitigate model-specific biasness [25].

In this data-driven study to determine the predictors, ML models, and risks associated with the incidence of HN lymphedema, we planned to overcome the multicollinearity and high dimensionality issues typical of dosimetric data by using the ensemble feature selection technique to first reduce the feature size and then subsequently select the non-collinear features to build models for both lymphedema prediction and risk analysis. Specifically, for ensemble feature selection, the filter and wrapper approaches were used first to select clinicopathologic and dosimetric features based on statistical information and recursive feature elimination techniques that are relevant to HN lymphedema. The relevant features were then used to train and tune ML models where the importance of each feature towards lymphedema incidence was scored and aggregated via a stacked feature importance plot. This reduced set of lymphedema-related features was (1) analyzed for multicollinearity where highly collinear features were further removed, leaving the remaining features as input (i.e., predictors) into the four ML models where their lymphedema prediction capability was evaluated; and (ii) build a competing risk model whereby the time-to-lymphedema and risk stratification could be performed using the cumulative incidence function (CIF) [26], [27] of features (i.e., predictors) significant of lymphedema. With the increasing desire to translate ML approaches for clinical usage, our unique use of ensemble feature selection and feature importance contributes towards improving model interpretability.

## Methods and materials

### Patient, clinicopathologic data, and endpoint

Patient data was collected as described in Rogacki et al. [28] In brief, in this IRB-approved retrospective study, 76 head and neck cancer patients consecutively treated by a single radiation oncologist from November 2016 to September 2019 were analyzed for lymphedema, following definitive- and adjuvant-intent radiotherapy. Clinical and pathological data, including the incidence of lymphedema, were extracted from electronic health records. Patients had a median follow-up time of 550 days after treatment with either external beam IMRT (n = 70) or proton radiotherapy (n = 6). Length of follow-up and time to lymphedema were determined from the end date of radiotherapy treatment. Incidence of external lymphedema was defined by records of lymphedema observed during physical exams or referrals to physical therapy for lymphedema. Internal lymphedema was defined by records of edematous laryngeal structures identified from laryngoscopies. Patients were censored at the time of the last oncology post-RT follow-up. Tumor recurrence and death without incidence of lymphedema were treated as competing events. Patient data were analyzed for external and internal lymphedema for the entire patient cohort and a subset of the patient population with an oropharyngeal primary (i.e., a total of four models). Oropharyngeal cancer was analyzed given that it is one of the most common forms of HNC.

### Dosimetric data from RT treatment plans

The contouring process and extraction of dosimetric data reported previously in Rocagki et al. [28] are summarized herein. CT images and patient treatment plans were imported into MIM (MIM Software Inc. Cleveland, OH), where volumes of interest were contoured. Following the consensus provided by Grégoire et al., [29] lymph node levels were contoured to include: bilateral lymph node levels (IB, IIA, IIB, III, IVA, IVB, V [posterior triangle group], VC [lateral supraclavicular group], VII [A & B combined]), bilateral parotid glands, midline lymph node levels IA and VIA, and the larynx. Composite structures comprising bilateral lymph node levels II-IV and bilateral lymph node levels IB-VII that are ipsilateral (IPSI) and contralateral (CONTRA) to the tumor were created. Bulky lymph node status was defined for tumors having N2c or greater, or N2 and greater for nasopharynx primaries. This status encompasses bilateral lymph node involvement, contralateral lymph node involvement, a lymph node greater than 6 cm, and extranodal extension (ENE). Dosimetric data for the corresponding structures were extracted from the 3D dose files using an open source code [30] to provide the respective dose-volume data feature.

### Selection of features associated with lymphedema incidence

**Fig. 1:**
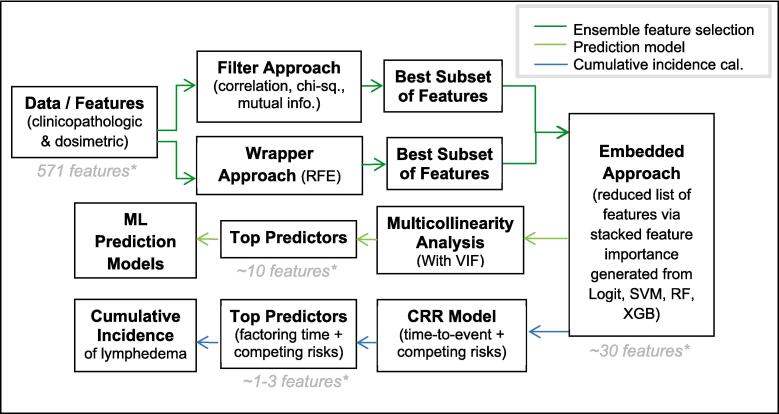
Pipeline to determine the predictors, prediction model and cumulative incidence of HN lymphedema via an ensemble feature selection process based on clinicopathologic and dosimetric data. (Abbreviations: ML = machine learning, RFE = recursive feature elimination, Logis = logistic model, SVM = support vector machine model, RF = random forest model, XGB = extreme gradient boost model, VIF = variance inflation factor, CRR = competing risk model).

Due to the high dimensionality of the data, a multi-stage ensemble feature selection process [20], [21], [22], [23], [24] involving the filter and wrapper methods was used to select top features associated with lymphedema incidence before feeding them into ML models (Fig. 1). The filter method, which evaluates the intrinsic properties of the data and measures their relevance via univariate test statistics, was used for feature selection given its independence from the classification/ML model. This method selected top features from the clinicopathologic and dosimetric datasets based on their Pearson correlation, mutual information, and chi-square test performance for the incidence of lymphedema. In the wrapper method, a recursive feature elimination (RFE) process trains a support vector machine (SVM) classifier to iteratively remove irrelevant features indicated by the weights of the SVM iteration. Common features from filter and wrapper methods were then fed into four ML models, where each feature's importance was generated. Feature importance provides an estimate of each feature's contribution toward each model's prediction of lymphedema. A stacked feature importance [24], [25] was used to rank the aggregated normalized importance of each feature derived from the four ML models [20], [21], [22], [23], [24]: logistic regression with Least Absolute Shrinkage and Selection Operator (LASSO) L1-regularization, SVM, Random Forrest (RF), and Extreme Gradient Boosting (XGBoost). These models were trained and tested using five-fold cross-validation and optimized via a hyper-parameter grid search approach (Table S1, Supplementary Material). The dataset was split into 80 %–20 % for training and testing the models, respectively. Feature imtportance scores were scaled between 0 and 1, with higher scores representing higher contributions.

### Lymphedema prediction models with low multicollinearity features

To assess the correlation strength between the features derived from the stacked feature importance, the variance inflation factor (VIF) was calculated [31]. Subsequently, approximately ten features were selected for the prediction models. This selection was based on the presence of low VIF scores, which indicate minimal multicollinearity.

Using the remaining features with low multicollinearity as inputs/predictors to the four ML models, the models’ lymphedema prediction capability was optimized with optimization objectives such as accuracy (ACC), F1-score, and area under the curve (AUC) [32]. The models’ ultimate capabilities in predicting lymphedema incidence were evaluated based on their performance on the test dataset. While accuracy is the measure of all true positives and true negatives, the F1-score is a harmonic mean of precision and recall, and the AUC is a single metric that combines both sensitivity and specificity in one number. Given that the AUC is less prone to instability due to an imbalanced dataset and the F1-score is sensitive to detecting differences in outcomes generated from the imbalanced dataset, both metrics were selected over the accuracy metric for the evaluation of the model's performance [32]. Feature selection and ML models were implemented using Python 3.8 and Scikit-Learn 1.0.2 library [33].

### Cumulative incidence of lymphedema with competing risks

The 30 top-ranked clinicopathologic and dosimetric features obtained from the initial stacked feature importance, along with censoring information and time-to-event were used as covariates in a multivariate Fine and Gray regression model [26], [27] where the significance of features to lymphedema incidence in the presence of competing risks (i.e., death and recurrence) were determined. Models were optimized by selecting features that minimize the Akaike information criterion (AIC) score in a forward selection approach [34], [35], [36]. Significance of variables (P value < 0.05) was determined using a Wald test. The cumulative incidence function (CIF) of lymphedema over time was calculated for the top significant predictors of external and internal lymphedema for the entire cohort and oropharynx subset following the well-established process provided by the others [36], [37]. Gray’s test was used to assess the significance of the difference between the CIFs of lymphedema versus recurrence/death [38]. Statistical analysis was performed with R 4.1.2 (The R Foundation, Austria).

### Assessment of lymphedema

Consistent with the National Comprehensive Cancer Network (NCCN) guidelines, [39] our routine clinical evaluations included head and neck examinations and flexible laryngoscopy at all follow-up appointments. These procedures were integral in assessing the presence and extent of lymphedema in patients’ post-treatment. External lymphedema occurrence was defined as lymphedema noted on physical exam or upon patient referral to physical therapy, which occurred only after lymphedema manifested, per our referral pattern. While established scales like the International Society of Lymphology staging system and the MD Anderson Lymphedema Scale exist, they were not routinely used in our clinical practice due to their novelty and lack of extensive supporting data. Consequently, we adopted a binary approach to classify lymphedema as either present or absent. Internal lymphedema assessment was conducted using laryngoscopy, with evaluations dichotomized into a binary outcome indicating the presence or absence of internal lymphedema. The assessment of internal lymphedema via flexible laryngoscopy was consistently performed by the same radiation oncologist. Additionally, evaluations by up to three experienced head and neck surgeons at our institution, particularly for patients undergoing adjuvant head and neck radiation therapy, were also included in our analysis. This approach provided a uniform method of assessment across the study, ensuring consistency in the evaluation of internal lymphedema. Following the assessment criteria for lymphedema, patients identified with external lymphedema were often referred for specialized physical therapy, such as complete decongestive therapy. It's important to note, however, that while the initiation of such therapy is crucial for prognosis, our dataset and analysis were not designed to predict the response to lymphedema therapy or to forecast the long-term prognosis of patients with respect to their lymphedema outcomes. Instead, our study focused on correlating various factors to predict the incidence of lymphedema at any point during the follow-up period. This approach aimed to identify potential risk factors associated with the development of lymphedema, rather than evaluating the effectiveness of therapeutic interventions or the progression of the condition post-diagnosis. The patients undergoing laryngectomy in our dataset all underwent total laryngectomy.

## Results

### Patient, clinicopathologic, and dosimetric data

Characteristics of our patient cohort were previously reported in Rogacki et al. [28] and reproduced in Table 1. Analysis of lymphedema following definitive-intent or adjuvant radiation treatment was conducted for the entire patient cohort (76 patients) and a subset of the cohort with oropharyngeal diseases (46 patients, 89 % were HPV-mediated). Most patients underwent definitive-intent radiation (63 %) and 72 % received concurrent chemotherapy with weekly cisplatin (86 %) or cisplatin every three weeks (7 %) as the most common regimen. The median follow-up time was 550 days (interquartile range of 276–––854 days) and the median number of days of radiation treatment was 46 and 48 for the entire and the orapharygeal patient cohort, respectively. A median EQD2 dose of 7000 cGy was received for the entire and orapharygeal patient cohort. From 65 clinicopathologic feature labels, 35 were used after dropping data that had more than 50 % missing values or were deemed redundant. There are 539 dosimetric features and two dependent outcomes—incidence of external and internal lymphedema.

**Table 1:** Patient, Tumor and Treatment Characteristics (Table reproduced from reference # 28).

Variable	All Patients (n = 76)	Oropharynx (n = 46)
**Patient characteristics**	N (%)	N (%)
Age at Completion of Treatment, median (range)	63 (19 – 86)	62 (30 – 77)
Sex		
Female	19 (25)	8 (17.4 %)
Male	57 (75)	38 (82.6 %)
Race		
African American	15 (19.7)	9 (19.6)
Asian	4 (5.3)	0
Caucasian	49 (64.5)	32 (69.6)
Hispanic	6 (7.9)	3 (6.5)
Not Specified	2 (2.7)	2 (4.3)
BMI, median (range)	24.3 (17.3 – 34.4)	24.3 (17.3 – 33.2)
Smoking Status		
Current	2 (2.6)	0
Former	44 (57.9)	25 (54.3)
Never	30 (39.5)	21 (45.7)
**Lymphedema Outcome**		
External Lymphedema	52 (68.4)	36 (78.3)
Internal Lymphedema	30 (39.5)	17 (37)
External & Internal Lymphedema	23 (30.3)	15 (32.6)
**Tumor Characteristics**		
Subsite		
Hypopharynx	2 (2.6)	–
Larynx	14 (18.4)	–
Nasopharynx	5 (6.6)	–
Oral Cavity	5 (6.6)	–
Oropharynx	46 (60.5)	46 (1 0 0)
Other (parotid, sinus & paranasal sinus)	4 (5.3)	–
Grouped^†^ T Stage		
Tis	2 (2.6)	0
T1	20 (26.3)	17 (37.0)
T2	22 (28.9)	14(30.4)
T3	13 (17.1)	5 (10.9)
T4	19 (25)	10 (21.8)
Consensus^‡^ T Stage		
T0	2	0
T1	20	17
T2	22	14
T3	13	5
T4	3	2
T4a	14	7
T4b	2	1
Grouped^††^ N Stage		
Group 0	16	5
Group 1	18	11
Group 2	9	7
Group 3	12	8
Group 4	21	15
Consensus^‡^ N Stage		
N0	16 (21.1)	5 (10.9)
N1	10 (13.2)	3 (6.5)
N2 (nasopharynx only)	2 (2.6)	
N2a	8 (10.3)	8 (17.4)
N2b	9 (11.8)	7 (15.2)
N2c	10 (13.2)	8 (17.4)
N3 (nasopharynx only)	1 (1.3)	0
N3a	0	0
N3b	20 (26.3)	15 (32.6)
Bulky Nodes*		
No	43 (56.6)	23 (50.0)
Yes	33 (43.4)	23 (50.0)
Location of lymph node metastases		
None	16 (21.1)	5 (10.9)
Unilateral	41 (53.9)	29 (63.0)
Bilateral	19 (25)	12 (26.1)
**Treatment Characteristics**		
**Surgery Variables**		
Surgical Resection		
No	48 (63.2)	29 (63.0)
Yes	26 (34.2)	17 (37.0)
Laryngectomy	2 (2.63)	0
Neck Dissection		
None	50 (65.8)	29 (63.0)
Unilateral (Ipsilateral)	20 (26.3)	4 (8.7)
Bilateral	6 (7.9)	13 (28.3)
Number of lymph nodes removed, average (IQR)	11.5 (0 – 20)	12.2 (0 – 21)
**Radiation Variables**		
Radiation Type		
Adjuvant	28 (36.8)	17 (37.0)
Definitive	48 (63.2)	29 (63.0)
Radiation treatment of neck		
None	6 (7.9)	0
Unilateral (Ipsilateral)	11 (14.5)	6 (13.1)
Bilateral	59 (77.6)	40 (87.0)
Radiation modality		
Photon	70 (92.1)	42 (91.3)
Proton	6 (7.9)	4 (8.7)
Radiation delivery		
2-field	6 (7.9)	0
SIB	57 (75)	38 (82.6)
Sequential	7 (9.2)	4 (8.7)
Proton	6 (7.9)	4 (8.7)
EQD2, median (range)	7000 (5200 – 7066)	7000 (5600 – 7066)
Days of radiation, median (range)	46 (28–70)	48 (37 – 54)
**Chemotherapy Variables**		
Induction chemotherapy		
No	71 (93.4)	42 (91.3)
Yes	5 (6.6)	4 (8.7)
Concurrent chemotherapy		
No	21 (27.6)	8 (17.4)
Yes	55 (72.4)	38 (82.6)
Adjuvant chemotherapy		
No	72 (94.7)	46 (1 0 0)
Yes	4 (5.3)	0

*Bulky nodes defined as N2c or greater, or N2 and greater for nasopharynx.

† Grouped T staging defined as T0 – T4, with substages T4a and T4b combined into single group T4.

†† Grouped N staging defined as:

0 = N0.

1 = N1 & N2a (includes nasopharynx).

2 = N2b.

3 = N2 (nasopharynx) and N2c.

4 = N3 (nasopharynx) and N3b.

‡ Consensus defined as highest T or N stage between clinical and pathologic staging.

IQR = Interquartile range.

SIB = simultaneous integrated boost.

### Features associated with lymphedema incidence

Table S2 (in the supplementay material) provides examples of the top ten features associated with the incidence of external lymphedema (entire cohort) selected using the filter method with correlation coefficient, mutual information, and chi-square test. Top features associated with the incidence of external and internal lymphedema are presented in Tables S3 and S4, respectively for the entire cohort and the oropharyngeal cohort (Tables S5 and S6, respectively) in the supplementary material.

### Predictors of lymphedema obtained with multicollinearity correction

**Fig. 2:**
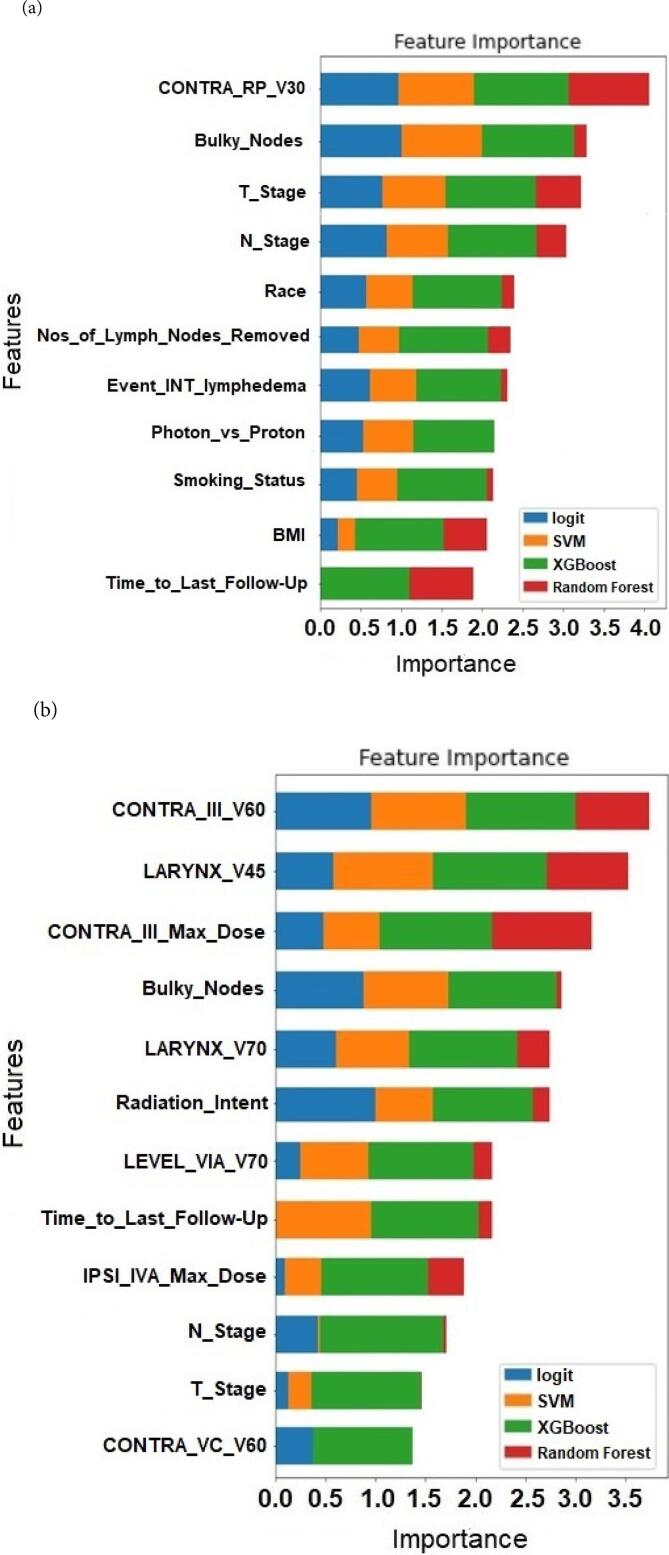

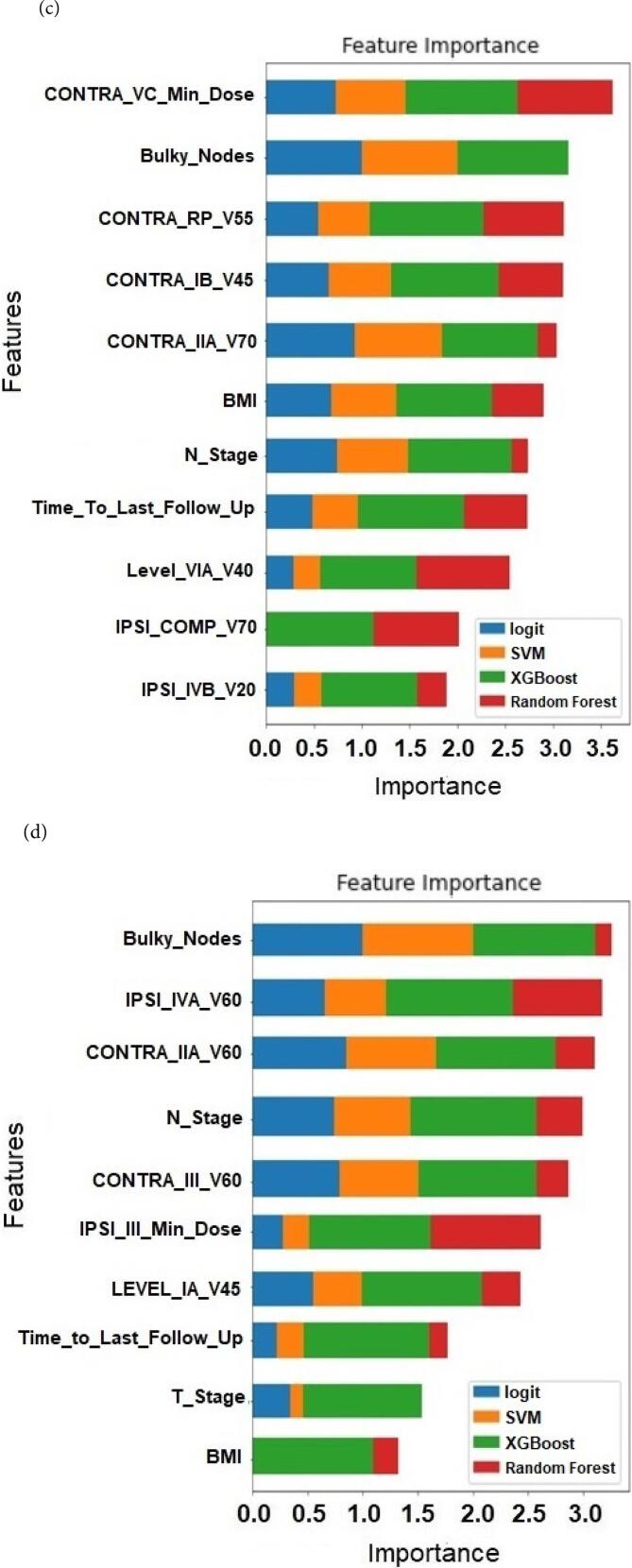
Stacked feature importance ranking determined by four ML models in predicting (a) external and (b) internal lymphedema (entire cohort); (c) external and (d) internal lymphedema (oropharyngeal cohort). (IPSI = ipsilateral; BILAT = bilateral; CONTRA = contralateral; COMP = composite; RP = retropharyngeal.).

After the removal of highly collinear features (high VIF scores) depicted in Tables S2-S5, Fig. 2 (a)–(d) depict the ranking (i.e., relative importance) of the top features (combined clinicopathologic and dosimetric features) used in optimizing the performance of the four ML models for external and internal lymphedema (entire and oropharyngeal cohorts) prediction respectively. These features are predictors for internal and external lymphedema for the ML models. Table S7 (in supplementary material) summarizes the VIF score for the final features depicted in Fig. 2. While most highly collinear features (VIF > 10) were removed, some features with high VIF were retained because they were deemed to be practically distinct from the other features. Their removal often resulted in a decrease in the ML models' performances. In comparing the four ML models for external and internal lymphedema prediction (entire and oropharyngeal cohorts), bulky lymph nodes at diagnosis, N Stage, and time to the last follow-up are the most common top predictors in all four cases; T Stage status and BMI are predictive of lymphedema in three of the cases, and contralateral level III V60 are predictive of internal lymphedema for both entire and oropharyngeal cohorts.

### Lymphedema prediction model evaluation

For the entire HN patient cohort, the top two ML models–XGBoost and random forest were capable of predicting (a) external lymphedema with an average F1-score and AUC of 84 ± 3 % and 79 ± 12 %; and (b) internal lymphedema with an average prediction performance of 64 ± 12 % and 78 ± 8 % for F1-score and AUC respectively. For the oropharyngeal patient cohort, XGBoost and random forest predicted (a) external lymphedema with an average F1-score and AUC of 89 ± 3 % and 71 ± 26 %; and (b) internal lymphedema with an average prediction performance of 67 ± 20 % and 84 ± 15 % for F1-score and AUC respectively. A comparison of the respective lymphedema prediction performance with the other two ML models (i.e., logistic regression and SVM) is provided in Table 2 and Figs. S1-S4 (in supplementary material).

**Table 2:** ML model prediction performance for external (EXT) and internal (INT) lymphedema for the two patient cohorts.

EXT lymphedema (entire cohort)	Mean			1 standard deviation		
	Accuracy	F1-score	AUC	Accuracy	F1-score	AUC
Logit	67.2	71.7	77.1	3.5	3.8	12.3
SVM	64.5	68.4	77.6	2.8	5.8	13
XGB	77.7	85.1	78.2	2.9	2.7	12.7
RF	74.9	83.3	79.1	5.3	3.8	11.2

INT lymphedema (entire cohort)						
Logit	69.9	59	74.5	8	17.4	9
SVM	63.3	55.8	70.7	7.4	18.1	9.2
XGB	76.3	66.9	79.5	6.7	13	7.8
RF	67.3	60.1	76	10	10.9	8.1

EXT lymphedema (oropharengeal cohort)						
Logit	58.7	68.1	70.9	10.9	10	20.2
SVM	76.2	86	68.2	3.1	3	22.5
XGB	84.9	91.3	67.6	4.9	2.6	28.3
RF	78.4	87.6	74.3	6	3.5	22.4
INT lymphedema (oropharengeal cohort)						
Logit	73.8	65.3	79.9	16.8	20.7	9.5
SVM	71.6	63.9	77.8	18.2	21.4	10.9
XGB	78.4	64.1	84.3	9.3	20	15.5
RF	80.7	70.4	83.7	12.3	20.4	15.3

Logit = Logistic regression, SVM = Support Vector Machine, XGB = Extreme Gradient Boosting,RF = Random Forest.

### Cumulative incidence of lymphedema with competing risks

Using lymphedema-associated features selected by the ensemble selection process (depicted in Tables S3-S6) along with time to lymphedema event, a multivariate competing risk regression analysis for the entire patient cohort found that the following features were significant and yielded an optimal model that converged: (external lymphedema) number of lymph nodes removed (*P* = 0.02), and maximum dose to the ipsilateral lymph node level VC (*P* = 0.01); and (internal lymphedema) maximum dose to the contralateral lymph node level III (*P* = 0.009) and percentage volume of the larynx receiving 60 Gy (*P* = 0.0129). Bulky nodes at diagnosis have a borderline statistical significance of *P* = 0.059 for external lymphedema.

For the oropharyngeal patient cohort, features that were significant and yielded optimal model include (external lymphedema) percentage volume of contralateral level IIA lymph node receiving 70 Gy (*P* = 0.00001); and (internal lymphedema) bulky lymph nodes (*P* = 0.045) and percentage volume of level IA lymph node receiving 45 Gy (*P* = 0.023).

**Fig. 3:**
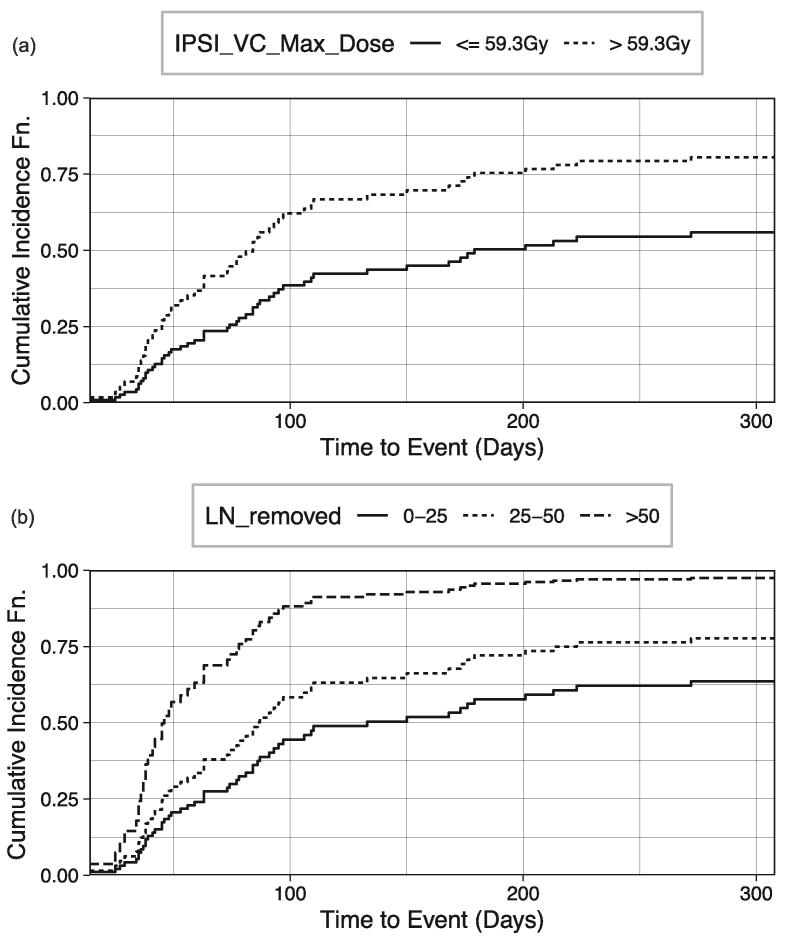
Cumulative incidence function of external lymphedema for the entire patient cohort stratified by low- and high-risk groups corresponding to (a) maximal dose of < 59.3 Gy and > 59.3 Gy received by the ipsilateral level VC lymph node respectively, and (b) the number of lymph nodes removed.

CIFs for all the significant features determined from the competing risk models were generated and used to separate patients into high and low-risk groups. Fig. 3 plots the estimated CIF of external lymphedema for the entire patient cohort stratified by low- and high-risk groups corresponding to (a) maximal dose of < 59.3 Gy and > 59.3 Gy received by the ipsilateral level VC lymph node, respectively, and (b) the number of lymph nodes removed. At 180 days, the cumulative incidence of external lymphedema with a maximal dose of < 59.3 Gy and > 59.3 Gy to the ipsilateral level VC lymph node were 50.3 % and 75.3 %, respectively (Gray's test, *P* = 0.01) (Fig. 3a). When 0–25, 26–50, and > 50 lymph nodes were removed, the increased risk of external lymphedema from 57.7 % to 72.1 % and 95.6 % respectively at 180 days is significant (Gray’s test, *P* = 0.01) (Fig. 3b).

**Fig. 4:**
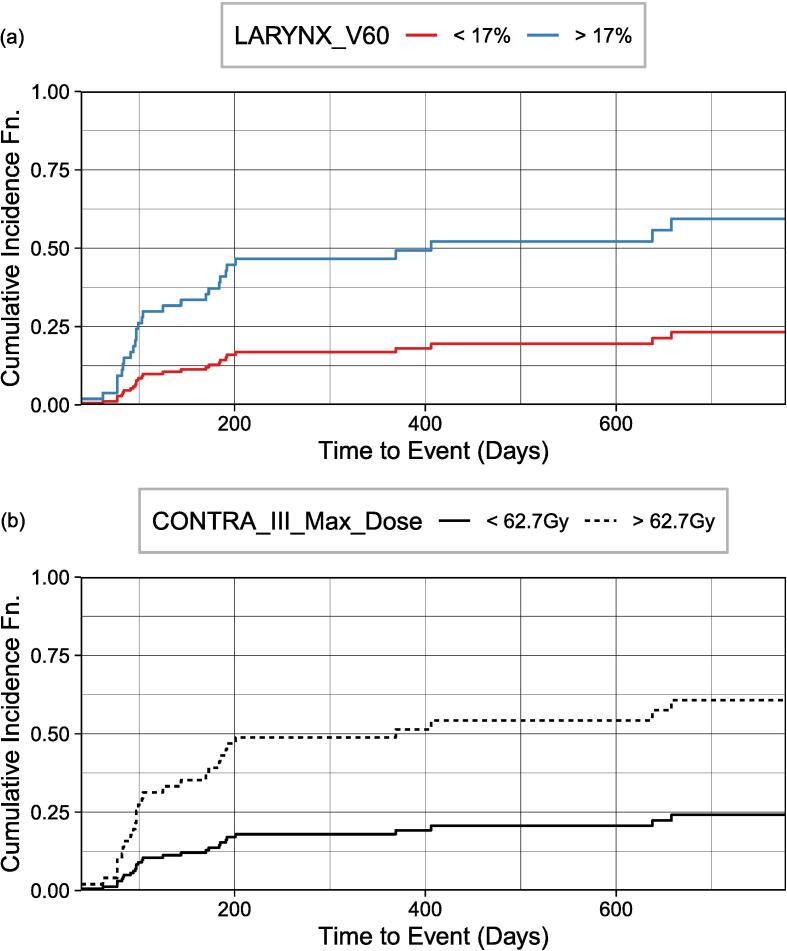
Cumulative incidence function of internal lymphedema for the entire patient cohort stratified by (a) the percentage volume of larynx receiving at least 60 Gy (V60) and (b) the maximum dose received by the contralateral level III nodes.

Fig. 4 plots the CIF of internal lymphedema for the entire patient cohort. At 180 days, cumulative incidences of 13.5 % and 39.1 % were significant (Gray’s test, *P* = 0.002) with < 17 % (the median volume) and > 17 % of the larynx receiving at least 60 Gy (V60) respectively (Fig. 4a). When stratifying the risk groups according to the maximum dose received by the contralateral level III nodes (Fig. 4b), cumulative incidences of 14.5 % and 41.1 % at 180 days for a maximum dose of < 62.7 Gy (median) and > 62.7 Gy were significant for the risk groups (Gray’s test, *P* = 0.002).

**Fig. 5:**
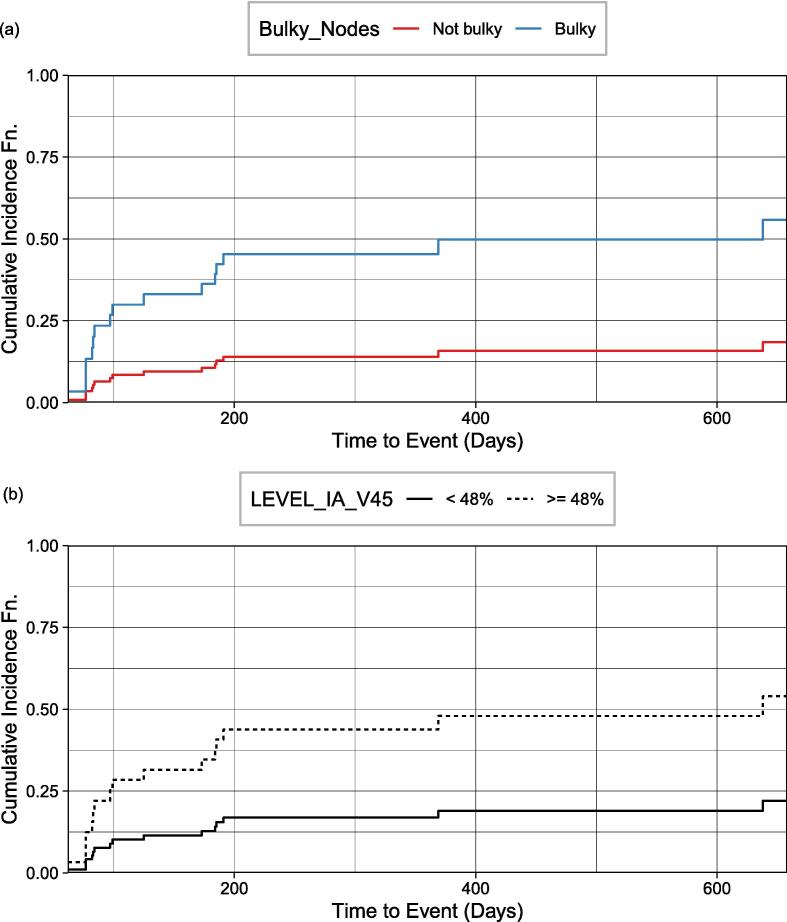
Cumulative incidence function of internal lymphedema for the oropharyngeal patient cohort stratified by (a) non-bulky versus bulky lymph nodes at diagnosis and (b) the volume of level IA nodes receiving 45 Gy.

For external lymphedema (oropharyngeal patient cohort), the CIF generated for 0 % (the median volume) and > 0 % of the volume of contralateral IIA level lymph nodes receiving 70 Gy were not statistically different for risk stratification (Gray’s test, *P* = 0.64). Fig. 5 plots the CIF of internal lymphedema for the oropharyngeal patient cohort. At 180 days, cumulative incidences of 11.7 % and 39.3 % for non-bulky and bulky lymph nodes at diagnosis are statistically significant for risk stratification (Gray’s test, *P* = 0.009) (Fig. 5a). Cumulative incidences of 14.1 % and 37.8 % associated with < 48 % and >= 48 % of level IA volume receiving 45 Gy respectively are significant for risk stratification (Gray’s test, *P* = 0.024) (Fig. 5b).

## Discussion

Our work had two distinct parts. The first part involved using an ensemble feature selection technique to select features associated with HN lymphedema and, in the process, reduce the size of the feature set. The second part used the reduced feature set to (i) optimize the lymphedema prediction performance of four ML models and (ii) build a competing risk model where censoring information was included to allow the prediction of time-to-lymphedema events and stratification of risk groups through the CIF generated. This multi-stage feature selection process, along with the use of stacked feature importance ranking not only mitigated issues associated with multicollinearity and high dimensionality that is common with radiotherapy dosimetric data but also enhanced the interpretability of the model in deriving the predictors and prediction models for lymphedema.

### Feature selection addressing high dimensionality, multicollinearity, and interpretability issues

The conventional approach of selecting features via a competing risk model often involves using a single integrated forward/backward elimination process to reduce the feature set before computing the CIF. Features were often selected based on the statistical significance and convergence of a specific risk model. The selection of features using a specific model is prone to model biasness that limits the generalizability of the predictors and models. Instead, our approach of using an ensemble feature selection technique allows inference and predictive techniques to systematically reduce the high dimensional feature set before input to ML and competing risk models. In addition, the focus of conventional survival analysis is to determine the top few features that could optimize the score (AIC or BIC) of the model, where the CIF would then be generated for those features. The relative importance of the features in associating with the outcome is often less obvious. One significant advantage of using our ensemble feature selection approach is the ability to visualize and interpret the features that were selected at different stages, as well as their associations with other similar features (via ranking) that are relevant to the incidence of lymphedema. This close ranking between similar features provides assurance and interpretability of the features selected.

### Predictors of lymphedema from ML and competing risk models

Clinicopathologic and dosimetric features (Tables S3-S6) obtained from the ensemble feature selection technique were used as input to both ML and competing risk models. While ML models require a more significant number of features to establish their prediction capability on unseen test data, the competing risk models, being inference in nature, searches within the existing dataset for a small, significant set of features to explain the occurrence of lymphedema. In addition, the ML models utilized the presence (or absence) of lymphedema as the modeling endpoint to generate the predictors of lymphedema while competing risk models take into account competing events (e.g., death, recurrence) and censoring time information in associating the features associated with lymphedema. A comparison showed that out of the seven lymphedema predictors selected from the competing risk models, six were predictors used in the ML models. The slight difference between the predictors selected by the models could be due to the sensitivity of existing data towards time-to-event and the presence of competing risks.

### Comparison with other studies that had both clinicopathologic and dosimetric features

We compared the list of lymphedema predictors from our study with Rogacki et al. which also has both clinicopathologic and dosimetric data features [28]. While we have used ensemble feature selection and stacked feature importance to systematically reduce the features before input to a competing risk model, Rogacki et al. rely on using a univariate analysis to select clinicopathologic features and iterative combinations with dosimetric features to identify the optimal model [28]. For external lymphedema (entire cohort), the number of lymph nodes removed appeared in both models as a significant feature for lymphedema. While the current risk model with ensemble feature selection indicated that the maximum dose to the ipsilateral level VC node was the other significant feature for lymphedema, the original study with univariate analysis noted contralateral RP V30 and bulky node status as a significant feature for lymphedema [28]. This may be due to the treatment of dosimetric features as continuous versus dichotomized variables and highlights model susceptibility to the character of data inputs. At the same time, a high dose to the ipsilateral lower neck could be a surrogate for bulky lymph nodes (defined as ≥ N2c disease, or ≥ N2 disease for nasopharyngeal primaries) at diagnosis as was hypothesized in the prior study [28]. In this study’s model, bulky node status was borderline significant (*P* = 0.059) for lymphedema occurrence.

For internal lymphedema (entire cohort), while the volume of the larynx receiving at least 60 Gy and maximum dose to the contralateral level III nodes were selected as prognostic features of lymphedema by our model, the volume of larynx receiving at least 45 Gy and ipsilateral level IVA maximum dose was selected by Rogacki et al. [28] Despite the differences in predictors of lymphedema, it should be noted that all the lymphedema predictors selected by their risk models were found in the initial list of reduced features obtained from our ensemble feature selection process, as depicted in Tables S3-S6.

For the oropharyngeal cohort, bulky lymph node involvement at diagnosis and level IA volume receiving > 45 Gy are statistically significant for the risk of lymphedema. This finding is unique since this patient population was not included in theirs [28] and other previous studies.

### Comparison with other lymphedema studies that had only clinicopathologic features

In a cross-sectional patient study by Deng et al., [8] six features were identified to be prognostic for the incidence of HN lymphedema, and they include tumor location, months after last HN cancer RT, total radiation dose, days of RT, number of treatment modalities, and radiation status of the surgical bed. Three of these features, months after the last HN cancer RT, total radiation dose, and days of RT (labeled as the time to last follow-up, total dose/EQD2, and days of radiation, respectively in our studies) were retained by our ensemble feature selection technique as relevant features for lymphedema in the various population cohorts:

• Time to the last follow-up for external and internal lymphedema (entire and oropharyngeal cohorts) (Tables S3-S6)
• Total dose/EQD2 for external lymphedema (entire and oropharyngeal cohort) (Tables S3 and S5)
• Days radiation for external lymphedema (entire cohort) and internal lymphedema (entire and oropharyngeal cohorts) (Tables S3, S4, S6)

However, only time to the last follow-up was subsequently selected as one of the predictors in our ML models for both external and internal lymphedema occurrence and the entire and oropharyngeal patient cohorts (Fig. 2a–d).

In the study by Tribius et al., [10] on patients who underwent surgical resection and chemoradiation, clinicopathologic predictors of lymphedema include: higher body mass index (BMI), extracapsular spread (ECE), linac-based IMRT (versus Tomotherapy®), the addition of chemotherapy, and extensive treatment to the bilateral versus ipsilateral neck with either neck dissection or RT. ECE spread corresponds to the bulky node status examined in our study, and surgical intervention is related to the number of lymph nodes removed. Except for the linac-based IMRT factor (tomotherapy not used in our practice), all remaining four features were identified as relevant features in our initial ensemble feature selection process (Tables S3-S6), and three (excluding the addition of chemotherapy) were ultimately selected as predictors of lymphedema in the subsequent ML and competing risk models: bulky lymph nodes at diagnosis (Fig. 2a, c, d, and Fig. 5), number of lymph nodes removed (Fig. 2a and Fig. 3), and BMI (Fig. 2a, 2c, d).

Both studies by Deng [8] and Tribius [10] revealed a similar trend where an increased number of treatment modalities (e.g., a combination of chemo-radiotherapy and surgery) would increase the risk of lymphedema. Sember et al. [39] reported that the odds of external lymphedema were increased with surgical resections of the primary tumor and/or neck dissection. In our study, we further quantified the impact of surgical interventions with the number of lymph nodes removed in the presence of radiation to specific lymphatic organs-at-risk.

In the study by Kim et al. [11] for lymphedema developing from nasopharyngeal carcinoma treatment, N-stage status was identified as a top predictor for lymphedema incidence. Similarly, N-stage status is one of the top predictors for external and internal lymphedema incidence (entire and oropharyngeal cohorts) in our ML predictive models (Fig. 2a–2d).

Although additional clinicopathologic predictors were reported in other studies, our current work—one of the first to include dosimetric features in the modeling, has shown that these clinicopathologic features were outweighed/replaced by some of the newly-added dosimetric data (as shown in Fig. 3, Fig. 4, Fig. 5).

Overall, the fact that our models generated several similar clinicopathologic features for HN lymphedema incidence compared to previous studies provides confidence that our data, feature selection, ML, and competing risk models were consistent in capturing some of the underlying features that were prognostic of HN lymphedema. Although our study derives dosimetric data from DICOM images, it is still considered a dose-volume histogram (DVH) based data mining approach. Future work could involve extracting image-based features, such as radiomics and deep learning features, to predict the incidence of HN lymphedema.

This work could impact clinical practice. Clinicopathologic features (e.g., number of lymph nodes removed and bulky lymph node involvement) may provide guidelines for close monitoring and early aggressive intervention to improve lymphedema outcomes [7], [40], [41], [42], [43], [44], [45]. While dosimetric features could provide guidelines to limit dose to the surrounding organ-at-risks when appropriate to do so (e.g., the maximum dose to the ipsilateral level VC lymph node (Fig. 3), larynx V60 and maximum dose to contralateral level III modes (Fig. 4), and level IA_V45 (Fig. 5).

In light of a recent study on the Lyman-Kutcher-Burman (LKB) model for predicting xerostomia [46], we reflect upon the distinctions between the LKB model and machine learning (ML) approaches in the context of lymphedema prediction. While the LKB model, primarily focusing on dose-volume histograms (DVHs), shows promise in xerostomia prediction, lymphedema in HN cancer presents a more complex challenge. Unlike xerostomia, which mainly results from radiation exposure to salivary glands, lymphedema's pathophysiology involves intricate disruptions in the lymphatic system, demanding an analysis beyond DVHs to encompass a range of clinical-pathologic features. In our study, we have identified several critical factors such as bulky lymph nodes at diagnosis, the number of lymph nodes removed, N Stage, T Stage status, time to the last follow-up, and BMI as significant predictors of lymphedema. These factors highlight the complexity of lymphedema prediction and the limitations of relying solely on DVH information. Given the multifaceted aspects of lymphedema, the nuanced approach of ML models, which integrates a broad range of clinical-pathologic and dosimetric factors, is essential, especially when compared to the LKB model's effectiveness in predicting xerostomia. Future research exploring or comparing the LKB model specifically for lymphedema prediction could yield further insights into optimizing prediction strategies.

### Limitations of our study

An important aspect of our study pertains to the choice of evaluation metrics. In this research, we primarily employed accuracy, AUC and F1 score to assess our machine learning model's performance in predicting HN lymphedema. Our decision to use these metrics was guided by their prevalent use in the field and their relevance to our study's design and objectives [47], [48]. However, this decision also opens a discussion about the potential limitations of current metrics and the suitability of alternative metrics such as log-loss and the Brier score. While accuracy, AUC and F1 score are effective in measuring the model's ability to discriminate between classes, they do not account for calibration — the extent to which predicted probabilities correspond to actual outcomes. In contrast, log-loss offers a deeper evaluation by considering predicted probabilities in addition to class discrimination. The Brier score, calculated as the sum of squared errors of probability estimates, provides insights into both model accuracy and confidence. As a proper scoring rule, its optimal value reflects perfect prediction and calibration. Considering these aspects, future research in this area might benefit from adopting a more diversified approach to model evaluation. By incorporating both traditional and proper scoring methods, researchers can achieve a more comprehensive assessment of model performance, especially in terms of calibration and confidence in predictions.

A potential limitation of our study is the small sample size. However, the robustness of our methodology helps mitigate this concern. Firstly, our multi-stage feature selection approach, which integrates ensemble feature selection, feature importance ranking, and multicollinearity analysis, is adept at handling high-dimensional data and ensures the identification of features essential for accurate lymphedema prediction. To enhance the reliability of our findings in light of the sample size, we employed cross-validation in training our models. This rigorous validation method significantly contributes to the robustness of our results, as evidenced by the promising AUC and F1 score averaging above 60 %, with our best-performing model achieving even higher accuracy. Additionally, we incorporated a competing risk model for further inference, pinpointing significant factors (P < 0.05) that influence lymphedema incidence. This cascaded methodology, fortified by cross-validation, bolsters the overall robustness and validity of our predictions despite the smaller sample. In addition, previous studies featuring a similar ratio of data to feature size have demonstrated that employing ensemble feature selection can yield robust features characterized by stable prediction performance [20], [21], [22], [23], [24], [49], [50], [51], [52]. These studies have demonstrated comparable results between predicted outcomes and experimental analysis for their application. For our application, significant overlap exists between the features identified in our study and those in prior lymphedema research, lending credibility to our study's rationale. This consistency in relevant features further supports the reliability of our findings within the broader context of lymphedema research. In summary, while our study navigates the complexities of clinical data analysis with a limited sample size, the methodologies and validation techniques employed aim to ensure the reliability and relevance of our findings. Future studies with larger datasets are encouraged to confirm and build upon our work.

Alongside our study's small sample size, several other limitations should be noted. Its retrospective nature could introduce biases, impacting the robustness of our findings. The wide age range of participants also presents a challenge, as it may affect the generalizability of our results across different age groups. Additionally, the use of non-standardized clinical data and varying classification scales might limit the consistency and comparability with other studies. These factors necessitate a cautious interpretation of our results and highlight the importance of future studies with standardized data collection, more homogeneous cohorts, and prospective designs to further validate and enhance our findings.

## Conclusions

By using an ensemble feature selection approach together with stacked feature importance and multicollinearity analysis for feature reduction, issues of multicollinearity and high dimensionality typical of RT dosimetric data were overcome, and the interpretability of the models’ outcome was enhanced. Our study is one of the first to report on the implementation and performance of ML models for external and internal lymphedema prediction for an entire population of HN cancers and separately for a cohort with oropharyngeal cancers. In our prediction models for external and internal lymphedema across entire and oropharyngeal cohorts, consistent top predictors included bulky lymph nodes at diagnosis, N Stage status, and time to the last follow-up. T Stage status and BMI were also predictive in three models. Specifically for internal lymphedema in both cohorts, contralateral level III V60 was identified as a significant predictor. In considering competing risks and time-to-event factors, we identified high-risk features for lymphedema. For external lymphedema (Fig. 3), significant predictors were the number of lymph nodes removed and a maximum dose exceeding 59.3 Gy to ipsilateral lymph node level VC. For internal lymphedema (Fig. 4), a high dose to contralateral level III (max. > 62.7 Gy) and larynx (V60 > 17 %) were key. Within the oropharyngeal cohort, factors indicating a high risk for internal lymphedema (Fig. 5) were the presence of bulky lymph nodes and high dose to level IA (V45 > 48 %). Stratification of risk by the top predictors could offer strategies for mitigating lymphedema, and treatment adaptation and rehabilitation could be initiated earlier. Given the lack of prior studies in using HN lymphedema as an endpoint for ML models, our models and findings serve as a basis for further research into predicting HN lymphedema incidence.

## CRediT authorship contribution statement

**P. Troy Teo:** Writing – original draft, Data curation, Software, Methodology, Investigation, Formal analysis, Visualization, Writing – review & editing. **Kevin Rogacki:** Data curation, Investigation, Formal analysis, Validation, Writing – review & editing. **Mahesh Gopalakrishnan:** Resources, Writing – review & editing, Data curation. **Indra J Das:** Writing – review & editing. **Mohamed E Abazeed:** Writing – review & editing. **Bharat B Mittal:** Writing – review & editing, Validation. **Michelle Gentile:** Conceptualization, Validation, Writing – review & editing, Supervision, Resources.

## Declaration of competing interest

The authors declare that they have no known competing financial interests or personal relationships that could have appeared to influence the work reported in this paper.
