# Supplementary material for: Determining risk and predictors of head and neck cancer treatment-related lymphedema: A clinicopathologic and dosimetric data mining approach using interpretable machine learning and ensemble feature selection
Source: Clin Transl Radiat Oncol. 2024 Feb 28;46:100747. doi: 10.1016/j.ctro.2024.100747 (PMC10915511; doi:10.1016/j.ctro.2024.100747)
Supplement: Supplementary data 1 [file mmc1.docx]

**Supplementary Materials**

Table S1. Details of grid search for hyper-parameter tuning

| Models | Hyper-parameters grid search |
| --- | --- |
| Support Vector Classifier | param_grid = {'C': array([0.001, 0.01, 0.1, 1, 10, 100, 1000] , 'kernel': ['linear', 'rbf']}) |
| Logistic Regression | param_grid = { 'C': array([0.001, 0.01, 0.1, 1, 10, 100, 1000], 'penalty': ['l1', 'l2'])} |
| XGBoost | param_grid={'colsample_bytree': [0.5, 0.75, 1], 'learning_rate': [0.3, 0.1, 0.03], 'max_depth': [2, 6, 12], 'min_child_weight': [1, 5, 15], 'n_estimators': [100], 'subsample': [0.5, 0.75, 1]} |
| RandomForest | param_grid={'n_estimators':n_estimators,'max_features':max_features, 'max_depth':max_depth,'min_samples_split':min_samples_split,'min_samples_leaf': min_samples_leaf, 'bootstrap': bootstrap} |

Hyper-parameter tuning performed according to:

<https://scikit-learn.org/stable/modules/generated/sklearn.model_selection.GridSearchCV.html>

Table S2 An example of top clinicopathologic and dosimetric features selected for external lymphedema (entire cohort) using the three statistical techniques of the filter method

| **Rank** | **Statistical Techniques of the Filter Method** | | |
| --- | --- | --- | --- |
|  | **Correlation Coefficient** | **Mutual Information** | **Chi-Square** |
| 1 | CONTRA RP V30 | Race | Bulky nodes |
| 2 | T Stage | Time to last follow up | T Stage |
| 3 | Bulky nodes | Bulky nodes | CONTRA RP V30 |
| 4 | N Stage | N Stage | N Stage |
| 5 | Nos. of lymph nodes removed | T Stage | Nos. of lymph nodes removed |
| 6 | Internal lymphedema | CONTRA RP V30 | Time to last follow up |
| 7 | Smoking status | Nos. of lymph nodes removed | Photon vs proton |
| 8 | Photon vs proton | BMI | Race |
| 9 | Race | Photon vs proton | BMI |
| 10 | BMI | Smoking status | Smoking status |

*Abbreviations:*

CONTRA = contralateral

RP = retropharyngeal

Vxx = volume receiving xx Gy

Table S3 List of 15 clinicopathologic and dosimetric features of relevance to external lymphedema incidence (entire cohort) selected by the ensemble feature selection method prior to multicollinearity analysis and pruning to remove highly collinear features

| **Rank** | **Clinicopathologic Features** | **Stacked Feature Importance** | **Rank** | **Dosimetric Features** | **Stacked Feature Importance** |
| --- | --- | --- | --- | --- | --- |
| 1 | T_Stage | 3.385 | 1 | CONTRA_RP_V30 | 3.891 |
| 2 | Bulky_Nodes | 3.289 | 2 | IPSI_IVB_Max_Dose | 3.776 |
| 3 | N_Stage | 3.203 | 3 | IPSI_IIB_V55 | 3.459 |
| 4 | Days_Radiation | 2.474 | 4 | IPSI_VC_Max_Dose | 3.377 |
| 5 | Number_of_lymph_nodes_removed | 2.366 | 5 | IPSI_IVB_V10 | 3.089 |
| 6 | BMI | 2.344 | 6 | IPSI_IIA_V55 | 3.067 |
| 7 | Time_to_Last_Follow_Up | 2.195 | 7 | IPSI_V_V10 | 2.773 |
| 8 | EQD2 | 2.174 | 8 | CONTRA_V65 | 2.700 |
| 9 | Photon_vs_Proton | 2.139 | 9 | CONTRA_IVB_V60 | 2.580 |
| 10 | Total_Dose | 2.138 | 10 | CONTRA_III_V65 | 2.470 |
| 11 | Smoking_Status | 2.099 | 11 | IPSI_III_V20 | 2.424 |
| 12 | Plan_Type | 1.883 | 12 | CONTRA_V_V65 | 1.903 |
| 13 | Subsite | 1.745 | 13 | CONTRA_IVA_V65 | 1.812 |
| 14 | Surgical_Resection | 1.735 | 14 | IPSI_IIB_V70 | 1.603 |

*Abbreviations:*

CONTRA = contralateral

IPSI = ipsilateral

RP = retropharyngeal

Vxx = volume receiving xx Gy

Table S4 List of 14 clinicopathologic and dosimetric features of relevance to internal lymphedema incidence (entire cohort) selected by the ensemble feature selection method prior to multicollinearity analysis and pruning to remove highly collinear features

| **Rank** | **Clinicopathologic Features** | **Stacked Feature Importance** |  | | **Rank** | | **Dosimetric Features** | | **Stacked Feature Importance** | |  |
| --- | --- | --- | --- | --- | --- | --- | --- | --- | --- | --- | --- |
| 1 | Radiation_Intent | 3.426 | |  | | 1 | | CONTRA_III_V60 | | 4.040 | |
| 2 | Bulky_Nodes | 3.302 | |  | | 2 | | LARYNX_V60 | | 3.489 | |
| 3 | Time_to_Last_Follow_Up | 3.033 | |  | | 3 | | LARYNX_V50 | | 3.409 | |
| 4 | N_Stage | 2.858 | |  | | 4 | | LARYNX_V55 | | 3.397 | |
| 5 | Age_at_Txt_Completion | 2.728 | |  | | 5 | | CONTRA_III_Max_Dose | | 3.330 | |
| 6 | Days_Radiation | 2.614 | |  | | 6 | | LARYNX_V65 | | 3.246 | |
| 7 | T_Stage | 2.588 | |  | | 7 | | LARYNX_V45 | | 2.992 | |
| 8 | Race | 2.343 | |  | | 8 | | LARYNX_V70 | | 2.889 | |
| 9 | Smoking_Status | 2.138 | |  | | 9 | | IPSI_COMP_Min.Dose | | 2.434 | |
| 10 | Event_EXT_lymphedema | 2.103 | |  | | 10 | | IPSI_IVA_Max_Dose | | 2.340 | |
| 11 | Re-CT? | 1.696 | |  | | 11 | | CONTRA_IVA_V60 | | 2.261 | |
| 12 | Number_of_lymph_nodes_removed | 1.600 | |  | | 12 | | CONTRA_VC_V60 | | 2.230 | |
| 13 | Gender | 1.424 | |  | | 13 | | CONTRA_III_V35 | | 2.203 | |
| 14 | Surgical_Resection | 1.385 | |  | | 14 | | LEVEL_VIA_V70 | | 2.104 | |

*Abbreviations:*

CONTRA = contralateral

IPSI = ipsilateral

RP = retropharyngeal

Vxx = volume receiving xx Gy

Table S5 List of 15 clinicopathologic and dosimetric features of relevance to external lymphedema incidence (oropharyngeal cohort) selected by the ensemble feature selection method prior to multicollinearity analysis and pruning to remove highly collinear features

| **Rank** | **Clinicopathologic Features** | **Stacked Feature Importance** |  | **Rank** | | **Dosimetric Features** | **Stacked Feature Importance** | |
| --- | --- | --- | --- | --- | --- | --- | --- | --- |
| 1 | T_Stage | 3.795 |  | 1 | CONTRA_VC_Min_ Dose | | 3.098 |  |
| 2 | Event_INT_lymphedema | 3.204 |  | 2 | CONTRA_IIA_V70 | | 3.069 |  |
| 3 | BMI | 3.087 |  | 3 | CONTRA_V70 | | 2.803 |  |
| 4 | Bulky_Nodes | 2.979 |  | 4 | CONTRA_IB_V45 | | 2.801 |  |
| 5 | Time_to_Last_Follow_Up | 2.774 |  | 5 | CONTRA_RP_V55 | | 2.684 |  |
| 6 | Number_of_lymph_nodes_removed | 2.670 |  | 6 | CONTRA_RP_V50 | | 2.674 |  |
| 7 | N_Stage | 2.618 |  | 7 | CONTRA_RP_V45 | | 2.548 |  |
| 8 | Induction_chemotherapy | 2.462 |  | 8 | CONTRA_RP_V40 | | 2.527 |  |
| 9 | Re-CT? | 2.329 |  | 9 | LEVEL_VIA_V40 | | 2.229 |  |
| 10 | Laterality_of_Primary | 2.264 |  | 10 | LEVEL_VIA_V70 | | 2.161 |  |
| 11 | Adjuvant_chemotherapy | 1.914 |  | 11 | IPSI_PAROTID_V70 | | 2.039 |  |
| 12 | Concurrent_chemoradiation | 1.899 |  | 12 | IPSI_IVB_V20 | | 1.743 |  |
| 13 | Total_Dose | 1.612 |  | 13 | BILAT_COMP_V75 | | 1.680 |  |
| 14 | EQD2 | 1.552 |  | 14 | IPSI_COMP_V70 | | 1.575 |  |
| 15 | Radiation_Intent | 1.197 |  | 15 | IPSI_IIA_V70 | | 1.343 |  |

Table S6 List of 15 clinicopathologic and dosimetric features of relevance to internal lymphedema incidence (oropharyngeal cohort) selected by the ensemble feature selection method prior to multicollinearity analysis and pruning to remove highly collinear features

| **Rank** | **Clinicopathologic Features** | **Stacked Feature Importance** | |  | **Rank** | | **Dosimetric features** | | | **Stacked Feature Importance** | | | | |  |
| --- | --- | --- | --- | --- | --- | --- | --- | --- | --- | --- | --- | --- | --- | --- | --- |
| 1 | Bulky_Nodes | 3.571 | |  | | 1 | | | CONTRA_IIB_V60 | | | 3.602 | |  |  |
| 2 | N_Stage | 3.404 | |  | | 2 | | | CONTRA_IIA_V60 | | | 3.380 | |  |  |
| 3 | Time_to_Last_Follow_Up | 2.846 | |  | | 3 | | | CONTRA_III_V60 | | | 3.188 | |  |  |
| 4 | T_Stage | 2.424 | |  | | 4 | | | IPSI_IVA_V60 | | | 3.103 | |  |  |
| 5 | BMI | 2.344 | |  | | 5 | | | CONTRA_IIA_V65 | | | 3.094 | |  |  |
| 6 | Age_at_Txt_Completion | 2.336 | |  | | 6 | | | IPSI_IVA_Max_Dose | | | 2.556 | |  |  |
| 7 | Days_Radiation | 2.286 | |  | | 7 | | | LEVEL_IA_V45 | | | 2.467 | |  |  |
| 8 | Number_of_lymph_nodes_removed | 2.152 | |  | | 8 | | | IPSI_III_Min_Dose | | | 2.388 | |  |  |
| 9 | Race | 2.063 | |  | | 9 | | | LEVEL_IA_V55 | | | 2.326 | |  |  |
| 10 | Smoking_Status | 1.896 | |  | | 10 | | | LEVEL_IA_V50 | | | 2.174 | |  |  |
| 11 | Photon_vs_Proton | 1.881 | |  | | 11 | | | CONTRA_V_V60 | | | 1.878 | |  |  |
| 12 | Plan_Type | 1.760 | |  | | 12 | | | CONTRA_V_V65 | | | 1.579 | |  |  |
| 13 | Re-CT? | 1.601 | |  | | 13 | | | LEVEL_IA_V60 | | | 1.538 | |  |  |
| 14 | Subsite | 1.594 | |  | | 14 | | | IPSI_VC_V60 | | | 1.147 | |  |  |
| 15 | Gender | 1.370 | |  | 15 | | | | CONTRA_VC_V75 | | | 1.124 |  |  |  |
|  |  | |  |  |  | | |  | | |  | | | | |

**Detecting multicollinearity using variance inflation factor (VIF)* analysis of top features/predictors of lymphedema selected by ML models.**

Table S7. VIF analysis of features/ predictors of lymphedema for external and internal lymphedema.

| **Entire Cohort** | | | | | | |
| --- | --- | --- | --- | --- | --- | --- |
| **External lymphedema** | | | |  | **Internal lymphedema** | |
| **VIF** | | | **Features/predictors** |  | **VIF** | **Features/predictors** |
| 18.8 | | | N_Stage |  | 24.5 | CONTRA_III_Max_Dose |
| 11.7 | | | Bulky_Nodes |  | 21.8 | N_Stage |
| 5.0 | | | T_Stage |  | 18.8 | IPSI_IVA_Max_Dose |
| 4.9 | | | CONTRA_RP_V30 |  | 14.1 | Bulky_Nodes |
| 4.1 | | | BMI |  | 12.7 | LARYNX_V45 |
| 4.0 | | | Time_to_Last_Follow_Up |  | 6.8 | T_Stage |
| 2.5 | | | Smoking_Status |  | 5.7 | Radiation_Intent |
| 2.2 | | | Event_INT_lymphedema |  | 4.6 | CONTRA_III_V60 |
| 1.7 | | | Number_of_lymph_nodes_removed |  | 4.1 | LARYNX_V70 |
| 1.7 | | | Race |  | 4.0 | Time_to_Last_Follow_Up |
| 1.2 | Photon_vs_Proton | | |  | 2.5 | LEVEL_VIA_V70 |
|  |  | | |  | 2.1 | CONTRA_VC_V60 |
| **Oropharyngeal Cohort** | | | | | | |
| 22.5 | | N_Stage | |  | 21.2 | N_Stage |
| 20.4 | | LEVEL_VIA_V40 | |  | 16.4 | CONTRA_III _V60 |
| 15.0 | | IPSI_IVB_V20 | |  | 14.5 | CONTRA_IIA_V60 |
| 15.0 | | Bulky_Nodes | |  | 13.9 | Bulky_Nodes |
| 12.3 | | CONTRA_IB_V45 | |  | 8.2 | IPSI_III_Min_Dose |
| 10.0 | | CONTRA_RP_V55 | |  | 4.4 | BMI |
| 7.5 | | CONTRA_VC_Min_Dose | |  | 4.2 | Time_to_Last_Follow_Up |
| 5.0 | | Time_to_Last_Follow_Up | |  | 3.5 | LEVEL_IA_V45 |
| 4.7 | | IPSI_COMP_V70 | |  | 2.7 | T_Stage |
| 4.4 | | BMI | |  | 1.8 | IPSI_IVA_V60 |
| 3.3 | | IPSI_PAROTID_V70 | |  |  |  |
| 3.1 | | CONTRA_IIA_V70 | |  |  |  |

*****These are the features with the smallest VIF for each of the patient cohorts and clinical endpoints. These features correspond to the minimal number of features needed for achieving an optimal level of predictive performance in our prediction models. Using these features as input to the inference model, features that are statistically significant to lymphedema will be selected by the competing risk model.

**External and internal lymphedema prediction performance of machine learning models for the entire and oropharyngeal cohorts.**

**
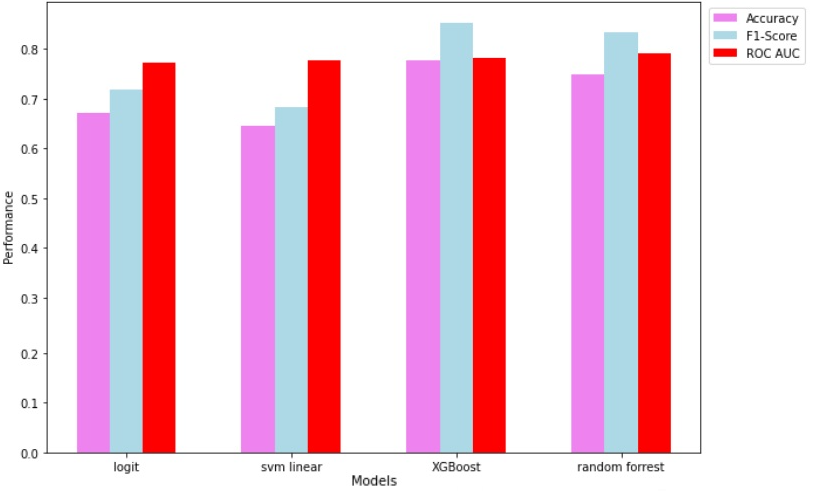
**

Figure S1 Prediction performances of ML models for external lymphedema (entire cohort).


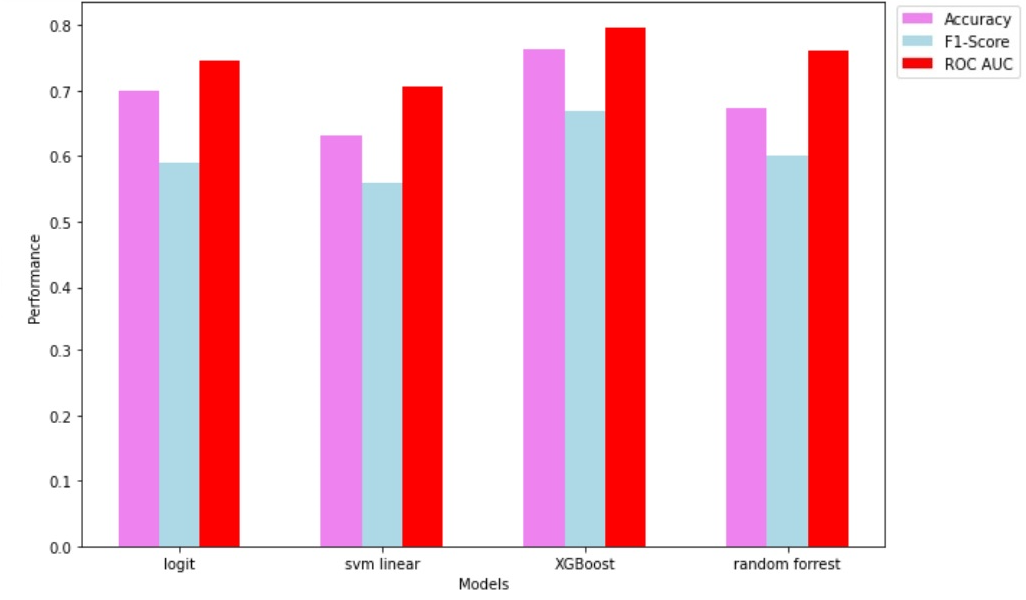


Figure S2 Prediction performances of ML models for internal lymphedema (entire cohort).

**
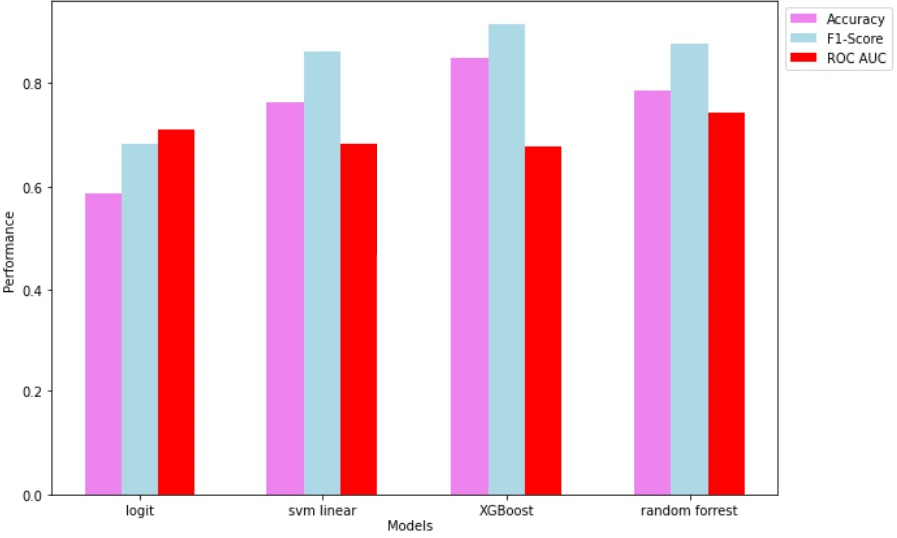
**

Figure S3 Prediction performances of ML models for external lymphedema (oropharyngeal cohort).

**
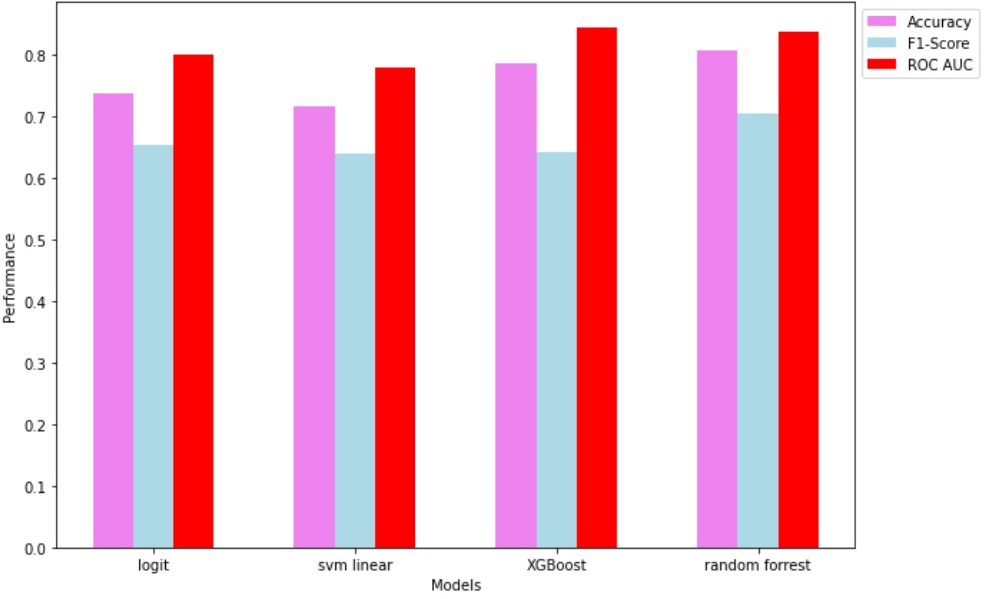
**

Figure S4 Prediction performances of ML models for internal lymphedema (oropharyngeal cohort).
